# Supplementary material for: SaPt-CNN-LSTM-AR-EA: a hybrid ensemble learning framework for time series-based multivariate DNA sequence prediction
Source: PeerJ. 2023 Oct 4;11:e16192. doi: 10.7717/peerj.16192 (PMC10559882; doi:10.7717/peerj.16192)
Supplement: Supplemental Information 2 [file peerj-11-16192-s002.pdf]

Supporting Information

SaPt-CNN-LSTM-AR-EA: A Hybrid Ensemble Learning Framework for Time Series-based Multivariate DNA Sequence Forecasting

Wu Yan [1,2,3,*], Li Tan [4], Li Meng-shan [4,*], Sheng Sheng [1, 3], Wang Jun [1, 3], Wu Fu-an [1, 3,*]

[1]School of Biotechnology, Jiangsu University of Science and Technology, Zhenjiang 212018, Jiangsu, China

[2] School of Mathematics and Computer Science, Gannan Normal University, Ganzhou 341000, Jiangxi, China;

[3] Sericultural Research Institute, Chinese Academy of Agricultural Sciences, Zhenjiang 212018, Jiangsu, China

[4] College of Physics and Electronic Information, Gannan Normal University, Ganzhou Jiangxi 341000, China

Correspondence to: Wu Yan (wuyan@gnnu.edu.cn) and Wu Fu-an (fuan_w@just.edu.cn)

Supporting Information

Supporting Information S1: Architecture

ARFIMA	p	d	q
ARFIMA1	2	0.137242	1
ARFIMA2	3	0.152319	3
ARFIMA3	0	0.103217	2
ARFIMA4	2	0.185938	1
ARFIMA5	1	0.187463	3
ARFIMA6	0	0.101779	1
ARFIMA7	0	0.186444	1
ARFIMA8	0	0.143275	3
ARFIMA9	3	0.174638	1
ARFIMA10	2	0.131683	3
ARFIMA11	1	0.10419	3
ARFIMA12	2	0.108895	2
ARFIMA13	0	0.10396	2
ARFIMA14	1	0.155748	3
ARFIMA15	2	0.113858	1
ARFIMA16	1	0.146806	3
ARFIMA17	0	0.121165	1
ARFIMA18	0	0.15631	2
ARFIMA19	2	0.136411	2
ARFIMA20	2	0.172277	2
ARFIMA21	1	0.167329	3
ARFIMA22	3	0.148079	3
ARFIMA23	1	0.190846	1
ARFIMA24	2	0.107327	3
ARFIMA25	1	0.113248	1
ARFIMA26	3	0.132143	3
ARFIMA27	0	0.177813	2
ARFIMA28	1	0.134341	3
ARFIMA29	0	0.159218	3
ARFIMA30	1	0.151746	3
ARFIMA31	1	0.155939	2
ARFIMA32	0	0.156593	1
ARFIMA33	2	0.168306	3
ARFIMA34	3	0.167236	2
ARFIMA35	2	0.192527	2

ARFIMA36	1	0.171254	3
ARFIMA37	0	0.141862	2
ARFIMA38	0	0.142844	1
ARFIMA39	0	0.171919	2
ARFIMA40	0	0.181297	2
ARFIMA41	2	0.165743	3
ARFIMA42	0	0.176136	3
ARFIMA43	3	0.177283	2
ARFIMA44	3	0.199419	2
ARFIMA45	1	0.104194	2
ARFIMA46	1	0.101288	2
ARFIMA47	2	0.122954	3
ARFIMA48	1	0.197995	1
ARFIMA49	2	0.130782	3
ARFIMA50	2	0.100007	3
ARFIMA51	2	0.127975	1
ARFIMA52	0	0.101963	2
ARFIMA53	3	0.111993	1
ARFIMA54	0	0.163274	3
ARFIMA55	1	0.173693	3
ARFIMA56	0	0.136911	1
ARFIMA57	1	0.150498	1
ARFIMA58	1	0.125376	3
ARFIMA59	0	0.112295	3
ARFIMA60	1	0.124829	3
ARFIMA61	0	0.18392	2
ARFIMA62	2	0.145758	2
ARFIMA63	3	0.15851	3
ARFIMA64	2	0.149439	3
ARFIMA65	1	0.175515	3
ARFIMA66	2	0.128742	1
ARFIMA67	3	0.106382	3
ARFIMA68	0	0.153435	1
ARFIMA69	3	0.116646	2
ARFIMA70	0	0.137962	1
ARFIMA71	3	0.122051	1
ARFIMA72	0	0.168472	3
ARFIMA73	2	0.135285	2
ARFIMA74	3	0.186815	3
ARFIMA75	2	0.127495	3
ARFIMA76	0	0.181889	1

Supporting Information S2: parameters

input_size	50
hidden_size	32
output_size	10
num_layers	3
bias	ture
batch_first	False
dropout	0
bidirectional	false
binary_dim	8
largest_number	2^binary_dim - 1
alpha	0.1
input_dim	50
hidden_dim	32
output_dim	10

Supporting Information S3: Codes

```
% implementation of RNN
clc
clear
close all
%% training dataset generation
binary_dim = 8;
largest_number = 2^binary_dim-1;
binary = cell(largest_number,1);
int2binary = cell(largest_number,1);
for i = 1:largest_number+1
binary{i} = dec2bin(i-1, 8);
int2binary{i} = binary{i};
end
%% input variables
alpha = 0.1;
input_dim = 2;
hidden_dim = 16;
output_dim = 1;
%% initialize neural network weights
synapse_0 = 2*rand(input_dim,hidden_dim) - 1;
synapse_1 = 2*rand(hidden_dim,output_dim) - 1;
```

```
synapse_h = 2*rand(hidden_dim,hidden_dim) - 1;
synapse_0_update = zeros(size(synapse_0));
synapse_1_update = zeros(size(synapse_1));
synapse_h_update = zeros(size(synapse_h));
%% train logic
for j = 0:19999
% generate a simple addition problem (a + b = c)
a_int = randi(round(largest_number/2)); % int version
a = int2binary{a_int+1}; % binary encoding
b_int = randi(floor(largest_number/2)); % int version
b = int2binary{b_int+1}; % binary encoding
% true answer
c_int = a_int + b_int;
c = int2binary{c_int+1};
% where we'll store our best guess (binary encoded)
d = zeros(size(c));
if length(d)<8
pause;
end
overallError = 0;
layer_2_deltas = [];
layer_1_values = [];
layer_1_values = [layer_1_values; zeros(1, hidden_dim)];
for position = 0:binary_dim-1
% X ------------------------> input
% sunapse_0 ----------------> U_i
% layer_1_values(end, :) ---> previous hidden layer （S(t-1)）
% synapse_h ----------------> W_i
% layer_1 ------------------> new hidden layer (S(t))
layer_1 = sigmoid(X*synapse_0 + layer_1_values(end, :)*synapse_h);
% layer_1 ------------------> hidden layer (S(t))
% output layer (new binary representation)
layer_2 = sigmoid(layer_1*synapse_1);
% did we miss?... if so, by how much?
layer_2_error = y - layer_2;
layer_2_deltas = [layer_2_deltas; layer_2_error*sigmoid_output_to_derivative(layer_2)];
overallError = overallError + abs(layer_2_error(1));
% decode estimate so we can print it out
d(binary_dim - position) = round(layer_2(1));
% store hidden layer so we can use it in the next timestep
layer_1_values = [layer_1_values; layer_1];
end
future_layer_1_delta = zeros(1, hidden_dim);
for position = 0:binary_dim-1
```

```
% a -> (operation) -> y, x_diff = derivative(x) * y_diff
X = [a(position+1)-'0' b(position+1)-'0'];
% prev_layer_1 ------------> (S(t-1))
layer_1 = layer_1_values(end-position, :);
prev_layer_1 = layer_1_values(end-position-1, :);
% error at output layer
layer_2_delta = layer_2_deltas(end-position, :);
output ，
% error at hidden layer
layer_1_delta = (future_layer_1_delta*(synapse_h') + layer_2_delta*(synapse_1')) ...
.* sigmoid_output_to_derivative(layer_1);
% let's update all our weights so we can try again
synapse_1_update = synapse_1_update + (layer_1')*(layer_2_delta);
synapse_h_update = synapse_h_update + (prev_layer_1')*(layer_1_delta);
synapse_0_update = synapse_0_update + (X')*(layer_1_delta);
future_layer_1_delta = layer_1_delta;
end
synapse_0 = synapse_0 + synapse_0_update * alpha;
synapse_1 = synapse_1 + synapse_1_update * alpha;
synapse_h = synapse_h + synapse_h_update * alpha;
synapse_0_update = synapse_0_update * 0;
synapse_1_update = synapse_1_update * 0;
synapse_h_update = synapse_h_update * 0;
if(mod(j,1000) == 0)
err = sprintf('Error:%s\n', num2str(overallError)); fprintf(err);
d = bin2dec(num2str(d));
pred = sprintf('Pred:%s\n',dec2bin(d,8)); fprintf(pred);
Tru = sprintf('True:%s\n', num2str(c)); fprintf(Tru);
out = 0;
size(c)
sep = sprintf('-------------\n'); fprintf(sep);
end
end

% implementation of LSTM
clc
clear
close all
%% training dataset generation
binary_dim = 8;
largest_number = 2^binary_dim - 1;
binary = cell(largest_number, 1);
for i = 1:largest_number + 1
binary{i} = dec2bin(i-1, binary_dim);
```

```
int2binary{i} = binary{i};
end
%% input variables
alpha = 0.1;
input_dim = 2;
hidden_dim = 32;
output_dim = 1;
%% initialize neural network weights
% in_gate = sigmoid(X(t) * U_i + H(t-1) * W_i) ------- (1)
U_i = 2 * rand(input_dim, hidden_dim) - 1;
W_i = 2 * rand(hidden_dim, hidden_dim) - 1;
U_i_update = zeros(size(U_i));
W_i_update = zeros(size(W_i));
% forget_gate = sigmoid(X(t) * U_f + H(t-1) * W_f) ------- (2)
U_f = 2 * rand(input_dim, hidden_dim) - 1;
W_f = 2 * rand(hidden_dim, hidden_dim) - 1;
U_f_update = zeros(size(U_f));
W_f_update = zeros(size(W_f));
% out_gate = sigmoid(X(t) * U_o + H(t-1) * W_o) ------- (3)
U_o = 2 * rand(input_dim, hidden_dim) - 1;
W_o = 2 * rand(hidden_dim, hidden_dim) - 1;
U_o_update = zeros(size(U_o));
W_o_update = zeros(size(W_o));
% g_gate = tanh(X(t) * U_g + H(t-1) * W_g) ------- (4)
U_g = 2 * rand(input_dim, hidden_dim) - 1;
W_g = 2 * rand(hidden_dim, hidden_dim) - 1;
U_g_update = zeros(size(U_g));
W_g_update = zeros(size(W_g));
out_para = 2 * rand(hidden_dim, output_dim) - 1;
out_para_update = zeros(size(out_para));
% C(t) = C(t-1) .* forget_gate + g_gate .* in_gate ------- (5)
% S(t) = tanh(C(t)) .* out_gate ------- (6)
% Out = sigmoid(S(t) * out_para) ------- (7)
% Note: Equations (1)-(6) are cores of LSTM in forward, and equation (7) is
% used to transfer hiddent layer to predicted output, i.e., the output layer.
% (Sometimes you can use softmax for equation (7))
%% train
iter = 99999; % training iterations
for j = 1:iter
% generate a simple addition problem (a + b = c)
a_int = randi(round(largest_number/2)); % int version
a = int2binary{a_int+1}; % binary encoding
b_int = randi(floor(largest_number/2)); % int version
b = int2binary{b_int+1}; % binary encoding
```

```
% true answer
c_int = a_int + b_int; % int version
c = int2binary{c_int+1}; % binary encoding
% where we'll store our best guess (binary encoded)
d = zeros(size(c));
if length(d)<8
pause;
end
% total error
overallError = 0;
% difference in output layer, i.e., (target - out)
output_deltas = [];
% values of hidden layer, i.e., S(t)
hidden_layer_values = [];
cell_gate_values = [];
% initialize S(0) as a zero-vector
hidden_layer_values = [hidden_layer_values; zeros(1, hidden_dim)];
cell_gate_values = [cell_gate_values; zeros(1, hidden_dim)];
% initialize memory gate
% hidden layer
H = [];
H = [H; zeros(1, hidden_dim)];
% cell gate
C = [];
C = [C; zeros(1, hidden_dim)];
% in gate
I = [];
% forget gate
F = [];
% out gate
O = [];
% g gate
G = [];
% start to process a sequence, i.e., a forward pass
% Note: the output of a LSTM cell is the hidden_layer, and you need to
% transfer it to predicted output
for position = 0:binary_dim-1
% X ------> input, size: 1 x input_dim
X = [a(binary_dim - position)-'0' b(binary_dim - position)-'0'];
% y ------> label, size: 1 x output_dim
y = [c(binary_dim - position)-'0']';
% use equations (1)-(7) in a forward pass. here we do not use bias
in_gate = sigmoid(X * U_i + H(end, :) * W_i); % equation (1)
forget_gate = sigmoid(X * U_f + H(end, :) * W_f); % equation (2)
```

```
out_gate = sigmoid(X * U_o + H(end, :) * W_o); % equation (3)
g_gate = tan_h(X * U_g + H(end, :) * W_g); % equation (4)
C_t = C(end, :) .* forget_gate + g_gate .* in_gate; % equation (5)
H_t = tan_h(C_t) .* out_gate; % equation (6)
% store these memory gates
I = [I; in_gate];
F = [F; forget_gate];
O = [O; out_gate];
G = [G; g_gate];
C = [C; C_t];
H = [H; H_t];
% compute predict output
pred_out = sigmoid(H_t * out_para);
% compute error in output layer
output_error = y - pred_out;
% compute difference in output layer using derivative
% output_diff = output_error * sigmoid_output_to_derivative(pred_out);
output_deltas = [output_deltas; output_error];
% compute total error
% note that if the size of pred_out or target is 1 x n or m x n,
% you should use other approach to compute error. here the dimension
% of pred_out is 1 x 1
overallError = overallError + abs(output_error(1));
% decode estimate so we can print it out
d(binary_dim - position) = round(pred_out);
end
% from the last LSTM cell, you need a initial hidden layer difference
future_H_diff = zeros(1, hidden_dim);
% stare back-propagation, i.e., a backward pass
% the goal is to compute differences and use them to update weights
% start from the last LSTM cell
for position = 0:binary_dim-1
X = [a(position+1)-'0' b(position+1)-'0'];
% hidden layer
H_t = H(end-position, :); % H(t)
% previous hidden layer
H_t_1 = H(end-position-1, :); % H(t-1)
C_t = C(end-position, :); % C(t)
C_t_1 = C(end-position-1, :); % C(t-1)
O_t = O(end-position, :);
F_t = F(end-position, :);
G_t = G(end-position, :);
I_t = I(end-position, :);
% output layer difference
```

```
output_diff = output_deltas(end-position, :);
% hidden layer difference
% note that here we consider one hidden layer is input to both
% output layer and next LSTM cell. Thus its difference also comes
% from two sources. In some other method, only one source is taken
% into consideration.
% use the equation: delta(l) = (delta(l+1) * W(l+1)) .* f'(z) to
% compute difference in previous layers. look for more about the
% proof at http://neuralnetworksanddeeplearning.com/chap2.html
% H_t_diff = (future_H_diff * (W_i' + W_o' + W_f' + W_g') + output_diff * out_para') ...
% .* sigmoid_output_to_derivative(H_t);
% H_t_diff = output_diff * (out_para') .* sigmoid_output_to_derivative(H_t);
H_t_diff = output_diff * (out_para') .* sigmoid_output_to_derivative(H_t);
% out_para_diff = output_diff * (H_t) * sigmoid_output_to_derivative(out_para);
out_para_diff = (H_t') * output_diff;
% out_gate diference
O_t_diff = H_t_diff .* tan_h(C_t) .* sigmoid_output_to_derivative(O_t);
% C_t difference
C_t_diff = H_t_diff .* O_t .* tan_h_output_to_derivative(C_t);
% % C(t-1) difference
% C_t_1_diff = C_t_diff .* F_t;
% forget_gate_diffeence
F_t_diff = C_t_diff .* C_t_1 .* sigmoid_output_to_derivative(F_t);
% in_gate difference
I_t_diff = C_t_diff .* G_t .* sigmoid_output_to_derivative(I_t);
% g_gate difference
G_t_diff = C_t_diff .* I_t .* tan_h_output_to_derivative(G_t);
% differences of U_i and W_i
U_i_diff = X' * I_t_diff .* sigmoid_output_to_derivative(U_i);
W_i_diff = (H_t_1)' * I_t_diff .* sigmoid_output_to_derivative(W_i);
% differences of U_o and W_o
U_o_diff = X' * O_t_diff .* sigmoid_output_to_derivative(U_o);
W_o_diff = (H_t_1)' * O_t_diff .* sigmoid_output_to_derivative(W_o);
% differences of U_o and W_o
U_f_diff = X' * F_t_diff .* sigmoid_output_to_derivative(U_f);
W_f_diff = (H_t_1)' * F_t_diff .* sigmoid_output_to_derivative(W_f);
% differences of U_o and W_o
U_g_diff = X' * G_t_diff .* tan_h_output_to_derivative(U_g);
W_g_diff = (H_t_1)' * G_t_diff .* tan_h_output_to_derivative(W_g);
% update
U_i_update = U_i_update + U_i_diff;
W_i_update = W_i_update + W_i_diff;
U_o_update = U_o_update + U_o_diff;
W_o_update = W_o_update + W_o_diff;
```

```
U_f_update = U_f_update + U_f_diff;
W_f_update = W_f_update + W_f_diff;
U_g_update = U_g_update + U_g_diff;
W_g_update = W_g_update + W_g_diff;
out_para_update = out_para_update + out_para_diff;
end
U_i = U_i + U_i_update * alpha;
W_i = W_i + W_i_update * alpha;
U_o = U_o + U_o_update * alpha;
W_o = W_o + W_o_update * alpha;
U_f = U_f + U_f_update * alpha;
W_f = W_f + W_f_update * alpha;
U_g = U_g + U_g_update * alpha;
W_g = W_g + W_g_update * alpha;
out_para = out_para + out_para_update * alpha;
U_i_update = U_i_update * 0;
W_i_update = W_i_update * 0;
U_o_update = U_o_update * 0;
W_o_update = W_o_update * 0;
U_f_update = U_f_update * 0;
W_f_update = W_f_update * 0;
U_g_update = U_g_update * 0;
W_g_update = W_g_update * 0;
out_para_update = out_para_update * 0;
if(mod(j,1000) == 0)
err = sprintf('Error:%s\n', num2str(overallError)); fprintf(err);
d = bin2dec(num2str(d));
pred = sprintf('Pred:%s\n',dec2bin(d,8)); fprintf(pred);
Tru = sprintf('True:%s\n', num2str(c)); fprintf(Tru);
out = 0;
sep = sprintf('-------------\n'); fprintf(sep);
end
end
```
