# Supplementary figures and images for: SaPt-CNN-LSTM-AR-EA: a hybrid ensemble learning framework for time series-based multivariate DNA sequence prediction
Source: PeerJ. 2023 Oct 4;11:e16192. doi: 10.7717/peerj.16192 (PMC10559882; doi:10.7717/peerj.16192)

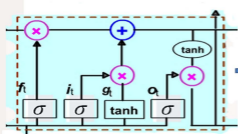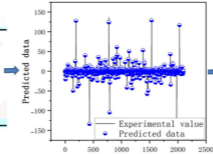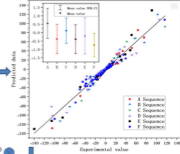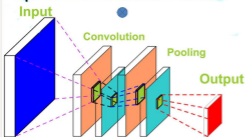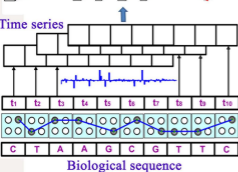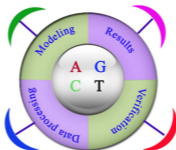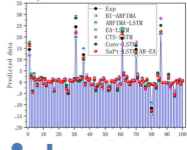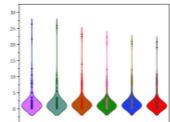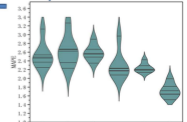

Supplement: Supplemental Information 3 [file peerj-11-16192-s003.pdf]
